# Supplementary figures and images for: pyPAGE: A framework for Addressing biases in gene-set enrichment analysis—A case study on Alzheimer’s disease
Source: PLoS Comput Biol. 2024 Sep 5;20(9):e1012346. doi: 10.1371/journal.pcbi.1012346 (PMC11421795; doi:10.1371/journal.pcbi.1012346)

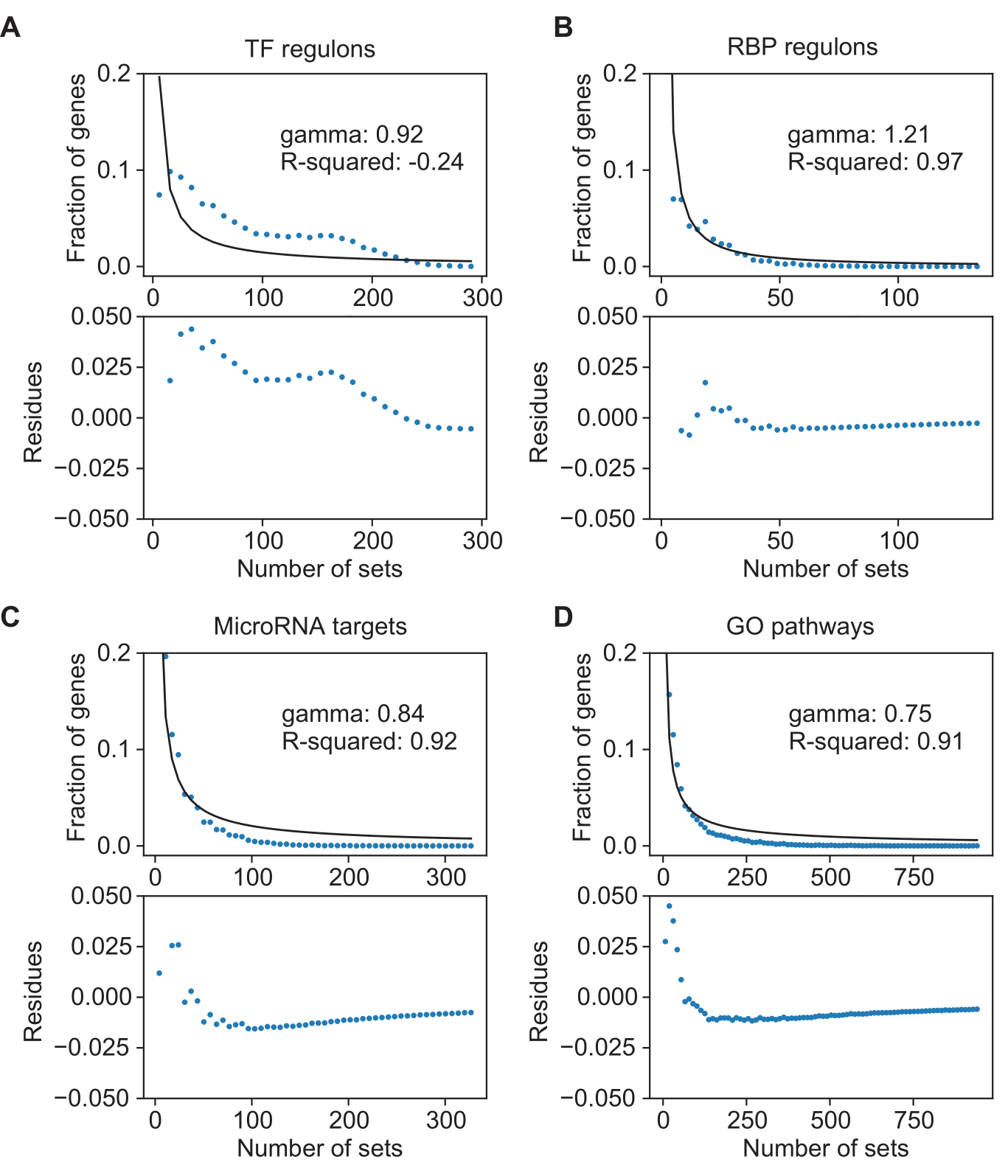

Supplement: S1 Fig — (A) Characteristics of a gene-set membership degree distribution in the TF regulons annotation. The top plot represents the observed distribution with a power law function being fit to it. We also report the gamma parameter of this distribution and the R2 The bottom plot represents the deviation of the observed distribution from the power law. (B) Similar representation for RBP regulons. (C) Similar representation for miRNA targets. (D) Similar representation for GO pathways. (TIFF) [file pcbi.1012346.s001.tiff]

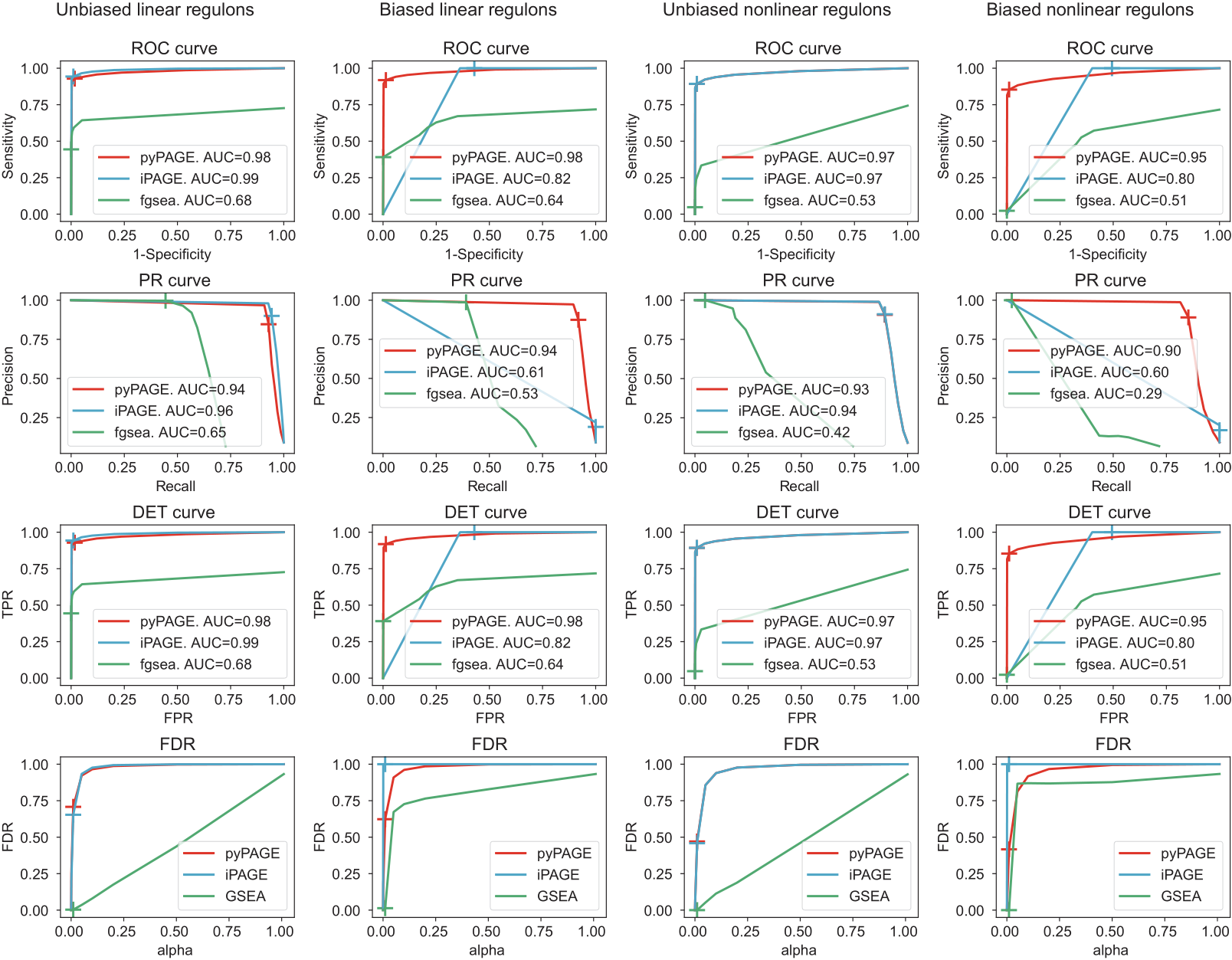

Supplement: S2 Fig — For comparison we used multiple metrics, results are presented as ROC, PR, DET curves and plot of dependency between alpha and FDR. (TIFF) [file pcbi.1012346.s002.tiff]

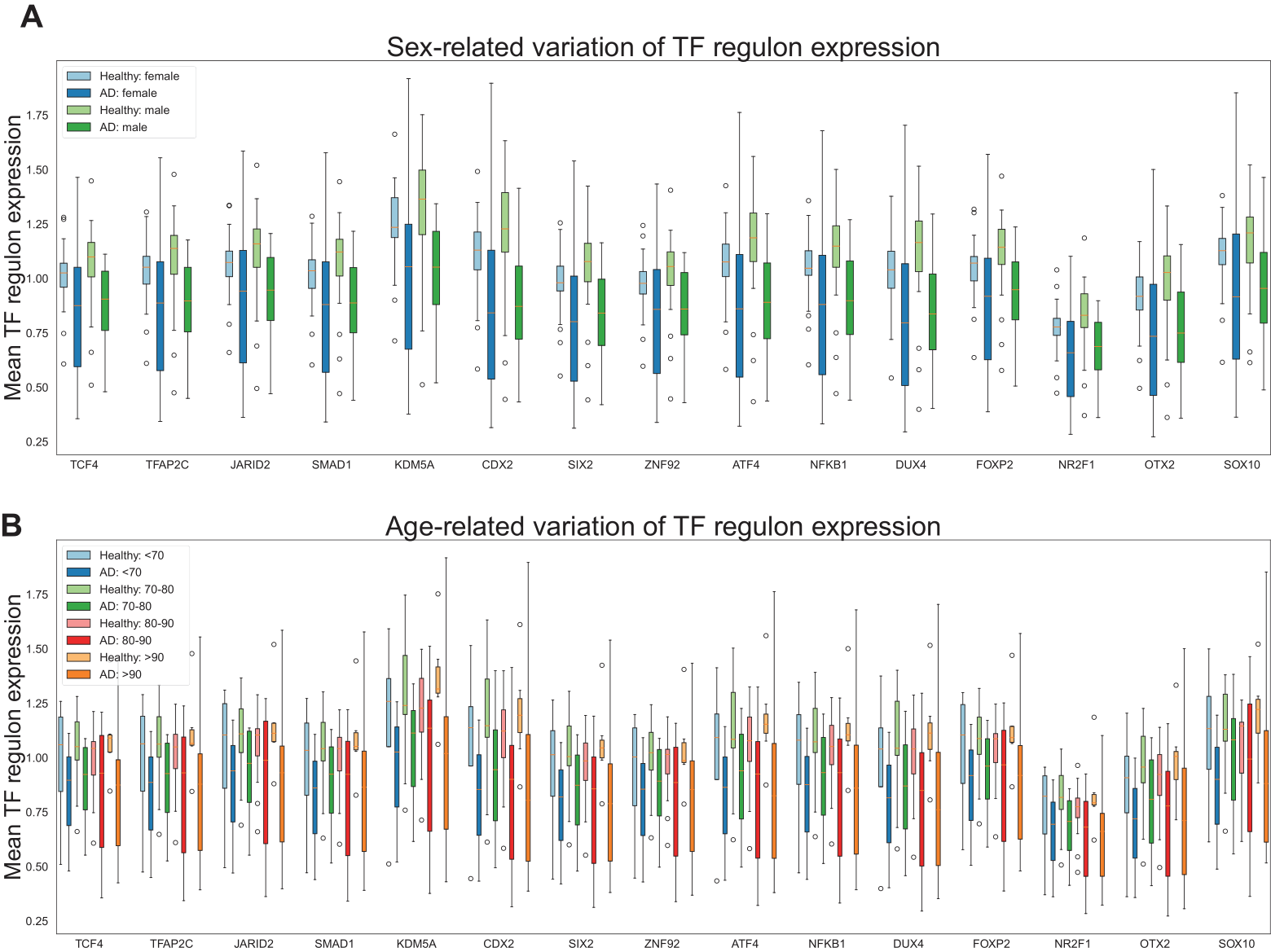

Supplement: S3 Fig — (A) Boxplots representing expression of TF regulons which were identified using pyPAGE in AD and non-AD samples from female and male donors. (B) Boxplots representing expression of the same TF regulons in AD and non-AD samples in different age cohorts. (TIFF) [file pcbi.1012346.s003.tiff]

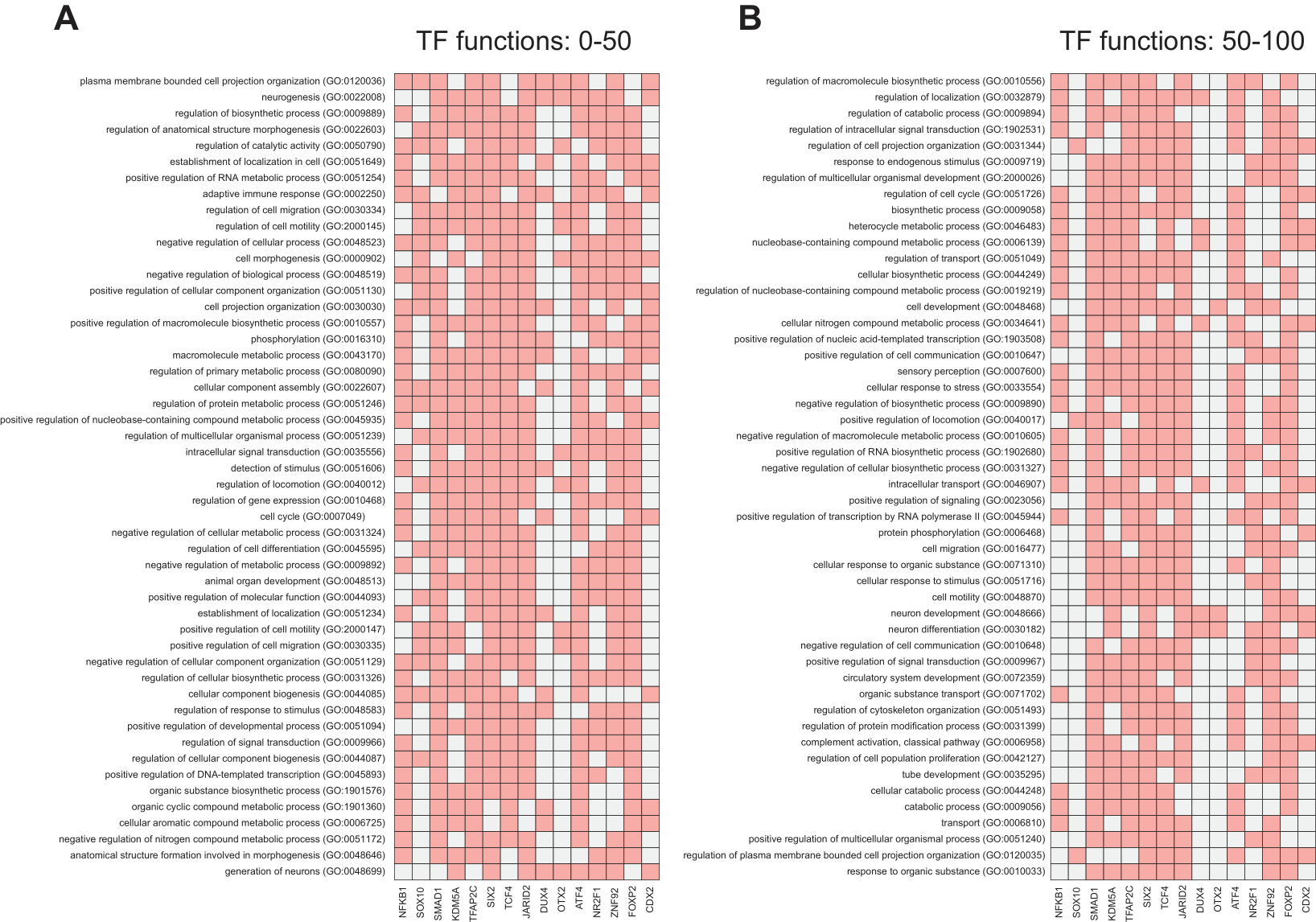

Supplement: S4 Fig — (A) Heatmap representing biological pathways that are enriched among targets of TFs we have identified in our analysis. Here we present top 50 pathways which are significantly enriched in most of the regulons excluding several generic terms, like “molecular process”. (B) This heatmap represents another 50 pathways significantly associated with the analyzed TFs. (TIFF) [file pcbi.1012346.s004.tiff]

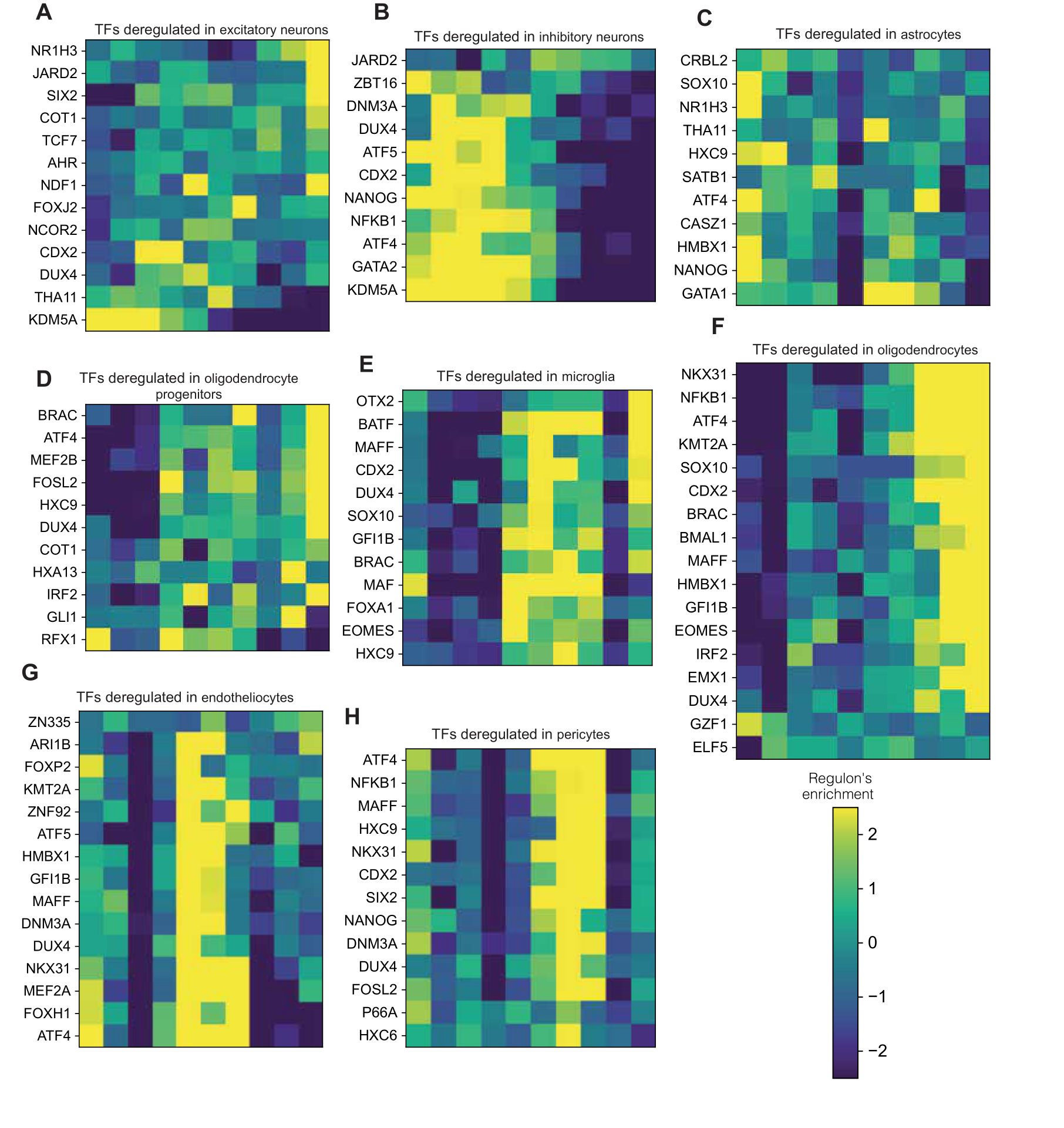

Supplement: S5 Fig — (A) Differential activity of TFs in excitatory neurons inferred using pyPAGE based on the analysis of single-cell RNA-seq data. (B) TF regulons deregulated in inhibitory neurons. (C) TF regulons deregulated in astrocytes. (D) TF regulons deregulated in oligodendrocyte progenitors. (E) TF regulons deregulated in microglia. (F) TF regulons deregulated in oligodendrocytes. (G) TF regulons deregulated in endotheliocytes. (H) TF regulons deregulated in pericytes. (TIFF) [file pcbi.1012346.s005.tiff]

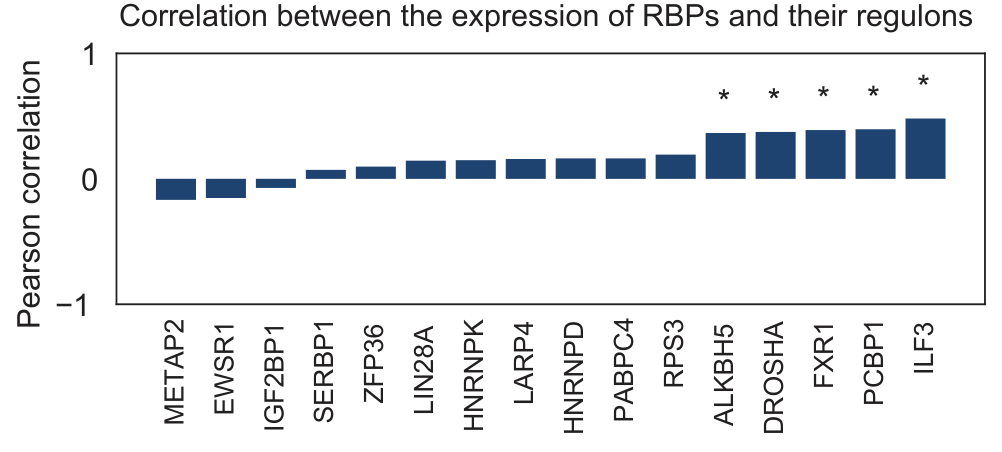

Supplement: S6 Fig — The barplot representing the correlations between the expression of RBPs and the average stability of their regulons. Asterix indicated significant correlation (p-value<0.05). (TIFF) [file pcbi.1012346.s006.tiff]

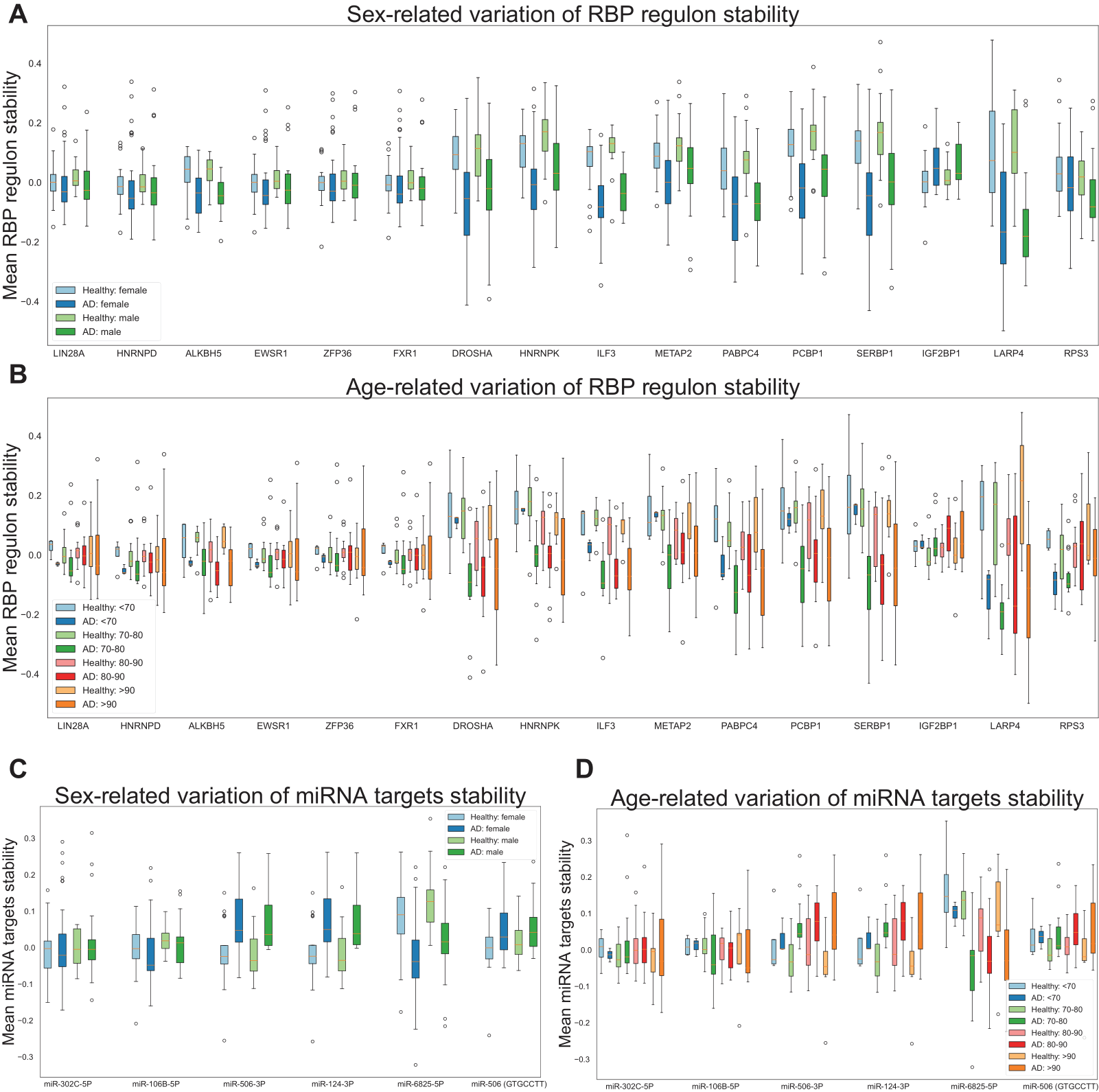

Supplement: S7 Fig — (A) Boxplots representing stability of RBP regulons which were identified using pyPAGE in AD and non-AD samples from female and male donors. (B) Boxplots representing stability of the same RBP regulons in AD and non-AD samples in different age cohorts. (C) Boxplots representing stability of miRNA targets within AD and non-AD samples from female and male donors. (D) Boxplots representing stability of miRNA targets within AD and non-AD samples from different age cohorts. (TIFF) [file pcbi.1012346.s007.tiff]

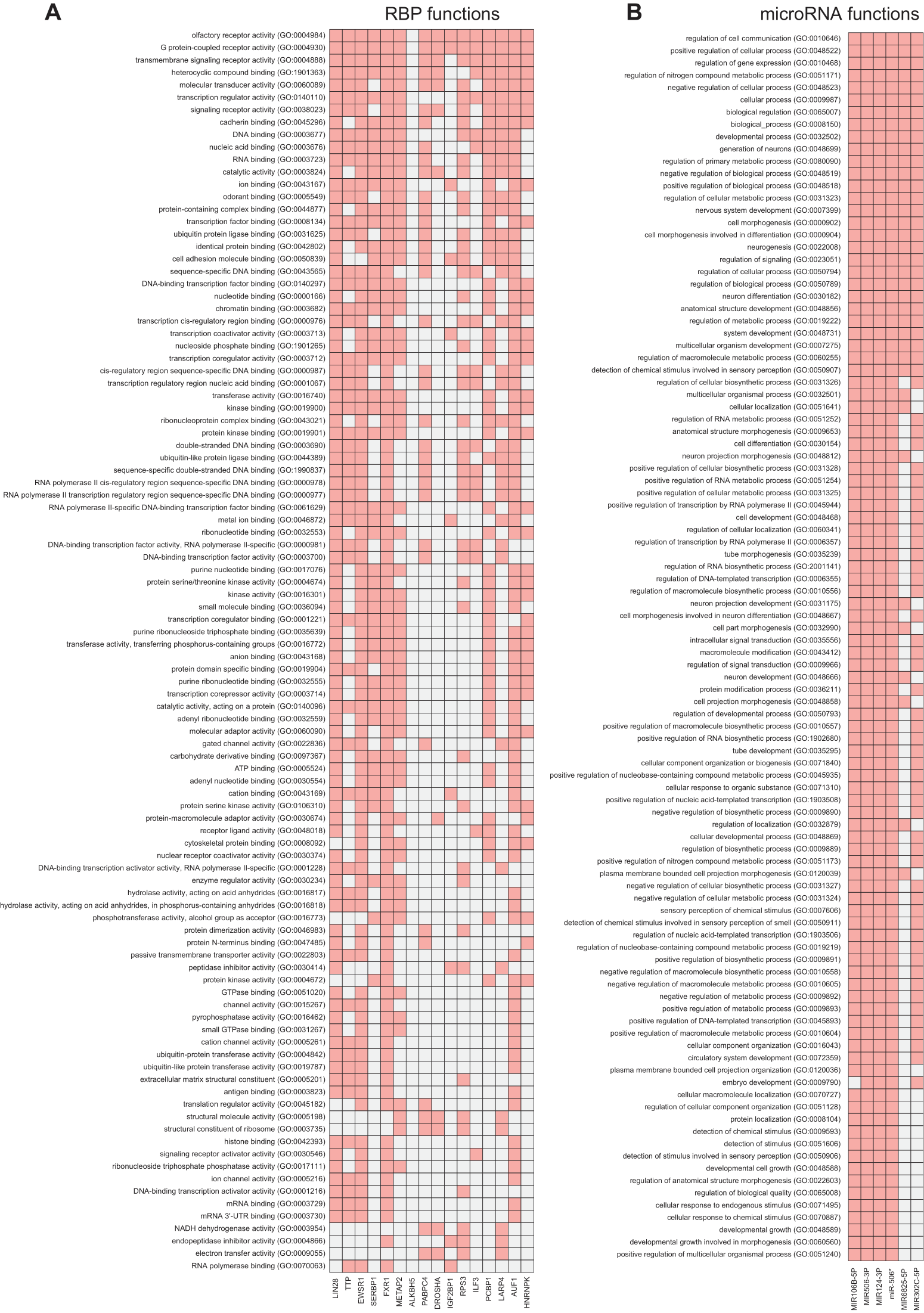

Supplement: S8 Fig — (A) Heatmap representing biological pathways that are enriched among targets of TFs we have identified in our analysis. Here we present only the top 100 pathways which are significantly enriched in most of the regulons. (B) Similar representation for miRNA targets. (TIFF) [file pcbi.1012346.s008.tiff]

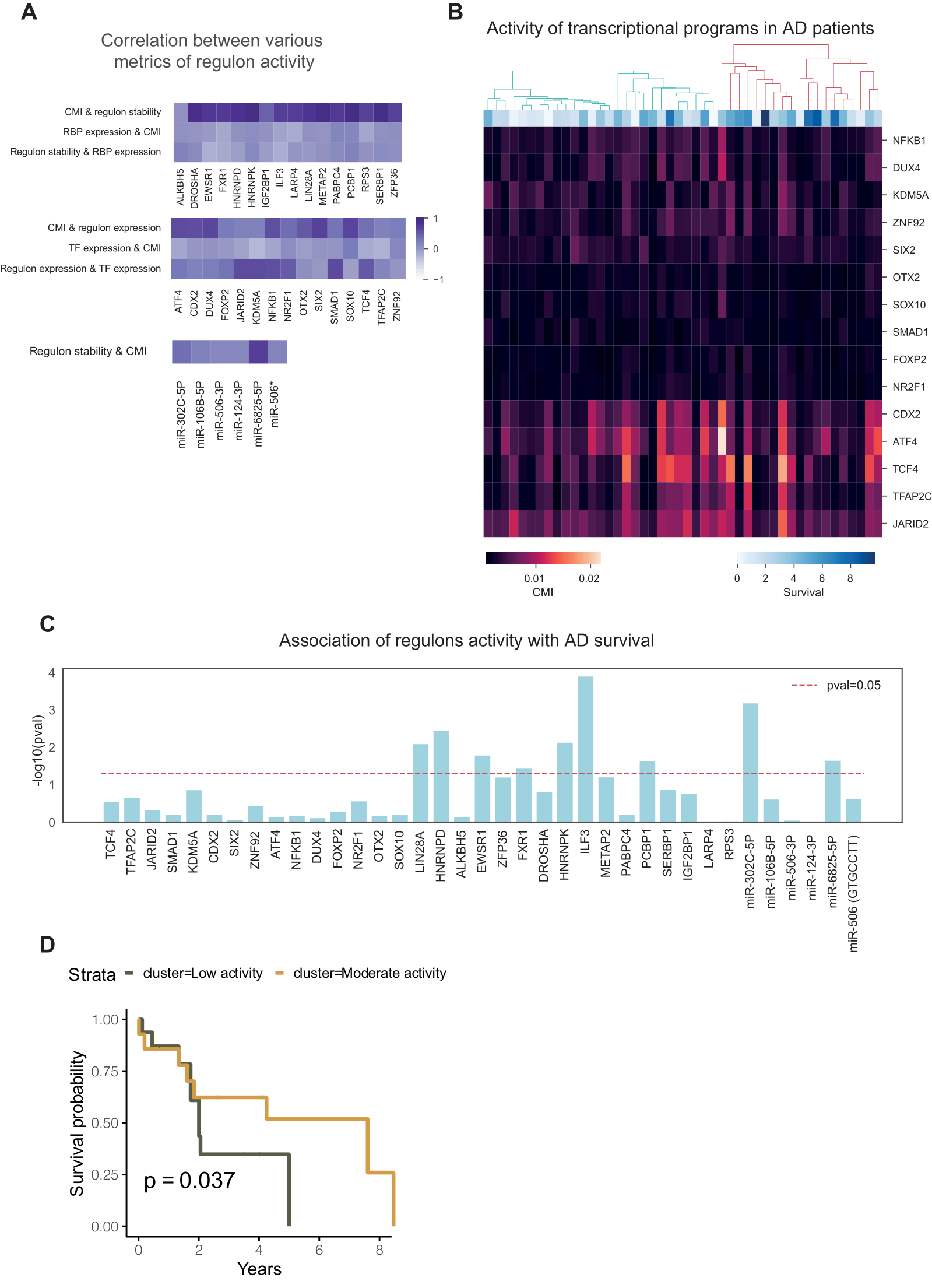

Supplement: S9 Fig — (A) Comparison of the similarity between various regulon activity metrics, namely conditional mutual information (CMI), mean regulon abundance and the expression of a factor itself. (B) Heatmap representation of activity of transcriptional regulons in different patients. (C) Bar Plot representing significance of association of activity of each previously identified regulon with patient’s survival. (D) Kaplan-Meier curve representing the difference in survival between two groups of patients stratified based on the activity of post-transcriptional regulons within the group with low RBP regulon activity. (TIFF) [file pcbi.1012346.s009.tiff]
