# Supplementary material for: pyPAGE: A framework for Addressing biases in gene-set enrichment analysis—A case study on Alzheimer’s disease
Source: PLoS Comput Biol. 2024 Sep 5;20(9):e1012346. doi: 10.1371/journal.pcbi.1012346 (PMC11421795; doi:10.1371/journal.pcbi.1012346)
Supplement: S1 Text — Table A in S1 Text. Differential enrichment of genomic variants in clusters identified based on RBP activity. Table B in S1 Text. Description of gene-set annotations. Table C in S1 Text. Summary of the pyPAGE experiments. (DOCX) [file pcbi.1012346.s010.docx]

# **pyPAGE: A Framework for Addressing Biases in Gene-Set Enrichment Analysis — A Case Study on Alzheimer’s Disease**

Artemy Bakulin^1^, Noam B Teyssier^2^, Martin Kampmann^2,7^, Matvei Khoroshkin^7,8,9,10*^, Hani Goodarzi^7,8,9,10*^

Affiliations:

^1^Faculty of Bioengineering and Bioinformatics, Lomonosov Moscow State University, Moscow, Russia

^2^Institute for Neurodegenerative Diseases, University of California, San Francisco, CA, USA.

^7^Department of Biochemistry and Biophysics, University of California, San Francisco, San Francisco, CA 94158, USA.

^8^Department of Urology, University of California, San Francisco, San Francisco, CA 94158, USA.

^9^Helen Diller Family Comprehensive Cancer Center, University of California, San Francisco, San Francisco, CA 94158, USA.

^10^Bakar Computational Health Sciences Institute, University of California, San Francisco, San Francisco, CA 94158, USA.

*Correspondence: [hani.goodarzi@ucsf.edu](mailto:hani.goodarzi@ucsf.edu) [matvei.khoroshkin@ucsf.edu](mailto:matvei.khoroshkin@ucsf.edu)

**Inventory**

**S1 Text Table A.**

**S1 Text Table B.**

**S1 Text Table C.**

**SUPPLEMENTARY TABLES**

**Table A. Differential enrichment of genomic variants in clusters identified based on RBP activity**

|  |  | **Position (hg37)** | **Associated genes** |
| --- | --- | --- | --- |
| **AD associated variants** | **Enriched in cluster A** | 10:130346154 | LINC02667 (upstream) |
|  |  | 1:207457845 | CD55 (upstream) |
|  |  | 5:57600044 | PLK2 (upstream) |
|  |  | 13:21712984 | SAP18 (upstream) |
|  |  | 14:29629456 | RNU11-5P, RNU6-864P (upstream) |
|  | **Enriched in cluster B** | 12:59221812 | LINC02388 (upstream) |
|  |  | 8:23571807 | STC1 (upstream) |
|  |  | 8:27487790 | SCARA3 (upstream) |
|  |  | 8:142611971 | - |
| **RBP associated variants** | **Enriched in cluster A** | 1:40045083 | PABC4 (upstream) |
|  | **Enriched in cluster B** | 3:180643165 | FXR1 (intron variant) |
|  |  | 12:50820467 | LARP4 (intron variant) |
|  |  | 12:50820468 | LARP4 (intron variant) |

**Table B. Description of gene-set annotations**

| **Name** | **Source** | **Number of gene-sets** | **Gene-set size** | **Gene-set membership** |
| --- | --- | --- | --- | --- |
| TF regulons | Vorontsov et al. (2018) | 495 | M=9495, SD=11600 | M=82, SD=63 |
| RBP regulons | ENCODE, POSTAR, Mireya Plass et al. (2017) | 344 | M=894, SD=966 | M=13, SD=14 |
| microRNA targets | MSigDB (MIR) | 2598 | M=156, SD=157 | M=24, SD=28 |

**Table C. Summary of the pyPAGE experiments**

| **Name** | **Annotation** | **Input** | **pyPAGE parameters** |
| --- | --- | --- | --- |
| TF regulons in bulk RNA-seq | TF regulons | Differential gene expression (source=MSBB [1], method=DESeq2 [2]) | alpha=0.01, redundancy_ratio=0.2, expr. bins=10, memb. bins=3 |
| RBP regulons in bulk RNA-seq | RBP regulons | Differential transcript stability (source=MSBB [1], method=REMBRANDTS [3] +t-test) | alpha=0.05, redundancy_ratio=0.3, expr. bins=10, memb. bins=3 |
| miRNA targets in bulk RNA-seq | microRNA targets | Differential transcript stability (source=MSBB [1], method=REMBRANDTS [3] +t-test) | alpha=0.01, redundancy_ratio=0.0, expr. bins=10, memb. bins=3 |
| TF regulons in single-cell RNA-seq | TF regulons | Differential gene expression (source=ROSMAP [4], method=t-test) | alpha=0.05, redundancy_ratio=0.0, expr. bins=10, memb. bins=3 |
| TF regulons in single-cell RNA-seq (novel) | TF regulons | Differential gene expression (source=ROSMAP [4], method=t-test) | alpha=0.01, redundancy_ratio=0.3, expr. bins=10, memb. bins=3 |
| TF regulons in spatial RNA-seq (Visium) | TF regulons | Differential gene expression (source=Chen et al. (2022) [5], method=Wilcoxon test) | alpha=0.05, redundancy_ratio=0.0, expr. bins=10, memb. bins=3 |
| RBP regulons in single-cell RNA-seq based on splicing rates | RBP regulons | Differential splicing rates (source=Morabito et al. (2021) [6], method=velovi [7] + t-test) | alpha=0.05, redundancy_ratio=0.0, expr. bins=10, memb. bins=3 |
| RBP regulons in single-cell RNA-seq based on degradation rates | RBP regulons | Differential degradation rates (source=Morabito et al. (2021) [6], method=velovi [7] + t-test) | alpha=0.05, redundancy_ratio=0.0, expr. bins=10, memb. bins=3 |
| miRNA targets in single-cell RNA-seq | microRNA targets | Differential degradation rates (source=Morabito et al. (2021) [6], method=velovi [7] + t-test) | alpha=0.05, redundancy_ratio=0.0, expr. bins=10, memb. bins=3 |

## **REFERENCE**

1. [Wang M, Beckmann ND, Roussos P, Wang E, Zhou X, Wang Q, et al. The Mount Sinai cohort of large-scale genomic, transcriptomic and proteomic data in Alzheimer’s disease. Sci Data. 2018 Sep 11;5(1):180185.](https://www.zotero.org/google-docs/?p9SNl3)
2. [Love MI, Huber W, Anders S. Moderated estimation of fold change and dispersion for RNA-seq data with DESeq2. Genome Biol. 2014 Dec 5;15(12):550.](https://www.zotero.org/google-docs/?p9SNl3)
3. [Alkallas R, Fish L, Goodarzi H, Najafabadi HS. Inference of RNA decay rate from transcriptional profiling highlights the regulatory programs of Alzheimer’s disease. Nat Commun. 2017 Oct 13;8(1):909.](https://www.zotero.org/google-docs/?p9SNl3)
4. [Mathys H, Davila-Velderrain J, Peng Z, Gao F, Mohammadi S, Young JZ, et al. Single-cell transcriptomic analysis of Alzheimer’s disease. Nature. 2019 Jun;570(7761):332.](https://www.zotero.org/google-docs/?p9SNl3)
5. [Chen S, Chang Y, Li L, Acosta D, Li Y, Guo Q, et al. Spatially resolved transcriptomics reveals genes associated with the vulnerability of middle temporal gyrus in Alzheimer’s disease. Acta Neuropathol Commun. 2022 Dec 21;10(1):188.](https://www.zotero.org/google-docs/?p9SNl3)
6. [Morabito S, Miyoshi E, Michael N, Shahin S, Martini AC, Head E, et al. Single-nucleus chromatin accessibility and transcriptomic characterization of Alzheimer’s Disease. Nat Genet. 2021 Aug;53(8):1143–55.](https://www.zotero.org/google-docs/?p9SNl3)
7. [Gayoso A, Weiler P, Lotfollahi M, Klein D, Hong J, Streets A, et al. Deep generative modeling of transcriptional dynamics for RNA velocity analysis in single cells [Internet]. bioRxiv; 2022 [cited 2023 Jan 29]. p. 2022.08.12.503709. Available from: https://www.biorxiv.org/content/10.1101/2022.08.12.503709v1](https://www.zotero.org/google-docs/?p9SNl3)
