# Supplementary material for: pyPAGE: A framework for Addressing biases in gene-set enrichment analysis—A case study on Alzheimer’s disease
Source: PLoS Comput Biol. 2024 Sep 5;20(9):e1012346. doi: 10.1371/journal.pcbi.1012346 (PMC11421795; doi:10.1371/journal.pcbi.1012346)
Supplement: Attachment — Submitted filename: review.docx [file pcbi.1012346.s014.docx]

Summary:

The authors provide compelling evidence for the need to correct for bias in pathway/GO enrichment analysis, by conditioning on biased genes. They compare to other tools using simulated data. They go on to demonstrate how their tool would be used in application to Alzheimer’s disease. Overall a well-written manuscript with a few issues with implementation and presentation, described below. The biggest issue is that the authors need to define more clearly what is required as input to their tool.

Major comments

1. Throughout the software and text, the term ‘expression’ is used. Please clarify. It seems that if the tool operates downstream of differential expression analysis, the relevant metric would be log fold change? The example simulated expression file provided on the github includes a column for genes, and a column for ‘expression’. Is this the average expression across all samples? The log fold change in expression between two groups? This needs clarification throughout the text and software.
2. Figure 1C, the workflow panel. It is not clear when and why one would need to use the right half of the workflow. Is it required? Please provide more motivation and explanation for this part.
3. Similarly, there is a line in the text that states ‘to analyze activity of post-transcriptional regulons one must take a step back to get the unbiased estimates of transcripts stability’. I don’t follow why this is a necessary step. Please motivate this more thoroughly.
4. Figure 1C ‘Gene counts’ is downstream of differential expression. The output of differential expression analysis is a gene-level summary of differential expression, with metrics such as log fold change, p-value, etc. Please clarify what is needed here. Related to point 1.
5. Please provide more information in the main text on how the bias was simulated.
6. Please provide more information on where the ‘known and putative interactions between regulators and genes’ come from, and how this information is used in the tool.
7. I do not understand Figure 2E/B (the heatmap with green squares). Please provide more explanation of how it was built, what it means, and what we can conclude from it. Or perhaps just remove it. Similar for Figure 4B.
8. Figure 5C: It is puzzling to me why the RBP regulon activity should be similar in healthy and AD shorter survival groups, but very different in AD longer survival. Can the authors provide some possible interpretation for this surprising result?
9. An extensive portion of the manuscript is spent describing how the pyPage tool is used to analyze Alzheimer’s disease datasets. It would be useful to include a comparison of results from traditional pathway analysis tools (e.g. GSEA, as described earlier in the text), to give the reader a sense of how different the results are using the new tool.

Minor comments

1. Define MSBB in introduction
2. Define what you mean by biological regulon.
3. Figure 1E: Please clarify what input modulation means. Does 0.2 mean 20% of the full data? So does 1.0 mean full signal? If so, then why are the two curves different at 1? I would expect them to be the same with no subsampling or damping.
4. Please add column labels to the heatmaps in Figures 2A, 3D, 4A,C
5. How were the cell types identified and annotated for the single cell analysis?
6. Legend in Figure 2 is not consistent with panel labels. B is E, C is B, etc.
7. Figure 5B not referenced in text. P-value in 5B is different from what is reported in the table in 5D- should they be the same? If not, please clarify the difference.

Software comments:

1. Load_annotation appears to switch genes and pathways, using example data
   a.

   b. I fixed it by setting ‘first_col_is_genes = True’, but should update your examples to be consistent with the data you provide
   c. Similar comment for the gene conversion- did not work with arguments on the github
2. Please include list of dependencies/requirements with versions
