# Supplementary material for: pyPAGE: A framework for Addressing biases in gene-set enrichment analysis—A case study on Alzheimer’s disease
Source: PLoS Comput Biol. 2024 Sep 5;20(9):e1012346. doi: 10.1371/journal.pcbi.1012346 (PMC11421795; doi:10.1371/journal.pcbi.1012346)
Supplement: Attachment — Submitted filename: pyPAGE-comments-2023.docx [file pcbi.1012346.s015.docx]

In this research article, Bakulin et al. tried to address biases in gene-set enrichment analysis which is a common practice for inferring genes’ function. Strengths of pyPAGE include: 1) it can also detect certain non-monotonic complex relationships such as dual regulators; 2) It does not depend on the distribution of the input data and can be applied to both discrete and continuous inputs. However, there are some major concerns that need to be carefully addressed:

1. Why to choose AD, especially considering that the iPAGE was developed by addressing cancers? Is the genetic risk landscape of AD that is different from cancers making an AD a unique challenge, more prune to biases? This shall be interesting to discuss, especially considering that most of the previous work were oriented to challenges posted by cancers, but what about AD, does it pose different challenges?
2. Along this line, How much customization need the pyPAGE be made to AD. Given any other diseases, is the method appliable and which hyperparameters need to be adjusted accordingly?
3. Pathway vs regulon: these two terms seem to be inter-changeably used but shall be clearly differentiated.
4. In abstract, “unbiased way” is a really bold claim. Can authors really avoid all the biases.
5. Along this line, authors claimed to be able to address the two biases, but it is not apparent to see how exactly this can be achieved.

First and foremost, I do not see why such what-authors-claimed-to-be “bias” is a bad thing, doesn’t this make more biological sense? Better annotated genes and abundant proteins are getting more weights. “differential expression of genes with specific functions provides stronger evidence for deregulation of its corresponding gene-sets than changes in expression of a more promiscuous gene.”

Second, if authors can convincingly address the above concerns, then we can talk about how exactly these two biases were tackled by proposed method. Using annotation bias as a covariate cannot completely address this type of bias and there is limitation. Besides, what about bias related to protein abundance difference? Authors shall really work out the details and explain to readers.

6. In Fig 1B, authors shall report p-values along with spearman correlation coefficients, which suggest significance of such correlations. The same issue applies to Fig 2 C and D, Fig S6 as well as all other correlation results.
7. Any stats results that involve multiple times of tests must be corrected for multiple-testings, due to the fact that they are prune to false positives. P<0.05 is not enough, and this issue applies to, but not limited to, Fig 2E, Fig 3CD.
8. “physical interactions for a given protein correlates with this protein abundance” and authors believed that this indicates structural biases. why this is the case? For sure, you can raise a few exceptions, but how can you conclude for sure that the majority shall not correlate?
9. What do you mean by multiple data modalities? Regardless of TF, RBP, and miRNA, aren’t they all table formats showing abundance information?
10. In Fig 5D, it is commendable to see the hint that stratification by regulon works, but the fact that cluster B seems to have greater variance may indicate that there is still significant heterogeneity leftover, therefore authors may try to break them into finer subgroups. Could it produce even better stratification?
11. The author compared the performance of pyPAGE, iPAGE, and fgsea on each simulated dataset. However, their performance on real-world data is still unclear and shall be further evaluated, and importantly against other popular enabling tools of the same kind.

Any regulons that the previous methods can identify but failed to be found by pyPAGE?

Also, when comparing against other methods, data shall be split into training set and testing set, and preferably bootstrap this split process for a large number of times and only report performance indices (with confident intervals) based on testing dataset.

12. In the analysis of single-cell data, for the differential expression of each cell type-specific gene, the author used estimated differential gene expression between two groups of samples. Would using marker genes specific to each cell type be more appropriate?
13. The author explored patterns in the single-cell data that had remained hidden in their analysis of bulk RNA-seq. Currently, spatial transcriptomics data related to Alzheimer’s disease have also been released, and I suggest considering adding an analysis or discussing relevant matters that specifically focused on spatial transcriptomics data. This way, it would encompass the mainstream transcriptional data involving gene-set enrichment analysis.
14. Some sentences need to be rephased, because it is hard to follow:

*For example, the observation that a protein that is only annotated as part of a few gene sets is differentially expressed narrows down the selection of potentially relevant pathways much more than a similar observation about a protein that is present in many gene sets.*

*the number of gene sets a gene is assigned to varies significantly among all genes.*

*which are by and large ignored*
