# Supplementary material for: pyPAGE: A framework for Addressing biases in gene-set enrichment analysis—A case study on Alzheimer’s disease
Source: PLoS Comput Biol. 2024 Sep 5;20(9):e1012346. doi: 10.1371/journal.pcbi.1012346 (PMC11421795; doi:10.1371/journal.pcbi.1012346)
Supplement: Attachment — Submitted filename: Rebuttal.docx [file pcbi.1012346.s016.docx]

**Reviewer #1**

**Comment 1.1**

Throughout the software and text, the term ‘expression’ is used. Please clarify. It seems that if the tool operates downstream of differential expression analysis, the relevant metric would be log fold change? The example simulated expression file provided on the github includes a column for genes, and a column for ‘expression’. Is this the average expression across all samples? The log fold change in expression between two groups? This needs clarification throughout the text and software.

**Response 1.1**

We thank the reviewer for their feedback. We defined the ‘expression’ column as we had done in our earlier work describing iPAGE[^1^](https://www.zotero.org/google-docs/?GNqNzS). We have now included this calculation of differential gene expression that we used for both the benchmarking and the analysis of Alzheimer's Disease in our manuscript:

(3) $DE=(1-p.value) * sign(log(fold change))$

This formula has also been included in the pyPAGE manual for easy reference.

This equation returns a signed metric of p-value, and in our earlier work we had shown that the resulting ranking of genes between similar is more robust compared to logFC. This is due to the tendency of logFC to over-exaggerate differences at low read counts/expression.

**Comment 1.2**

Figure 1C, the workflow panel. It is not clear when and why one would need to use the right half of the workflow. Is it required? Please provide more motivation and explanation for this part.

**Response 1.2**

We thank the reviewer for the comment. We have revised the schematic of the pyPAGE pipeline to more effectively illustrate the analytical logic. The updated design clearly demonstrates that pyPAGE can be used to analyze changes in gene expression as a whole (left) or specifically post-transcriptional levels (right). It also shows which set of preprocessing toolkits should be used to get appropriate inputs for the analysis at each level.

**Comment 1.3**

Similarly, there is a line in the text that states ‘to analyze activity of post-transcriptional regulons one must take a step back to get the unbiased estimates of transcripts stability’. I don’t follow why this is a necessary step. Please motivate this more thoroughly.

**Response 1.3**

We thank the reviewer for this comment. We have provided a rationale in the main text for why the analysis of post-transcriptional regulation should be performed using differential stability rather than differential expression measurements. In short, differential gene expression primarily reflects changes in transcription, while RBPs and microRNAs influence the stability of transcripts. Therefore, to analyze the deregulation of their target transcripts, it is necessary to obtain estimates of changes in transcript stability.

As described in our earlier work introducing REMBRANDTS[^2^](https://www.zotero.org/google-docs/?I18FaL), we described the need for these added steps to estimate changes in RNA stability. In short, gene expression as a whole is more influenced by transcriptional regulation rather than post-transcriptional control. Therefore, it is imperative that we zero in on the component of gene expression changes that is due to RNA stability to boost the signal-to-noise for post-transcriptional regulons.

**Comment 1.4**

Figure 1C ‘Gene counts’ is downstream of differential expression. The output of differential expression analysis is a gene-level summary of differential expression, with metrics such as log fold change, p-value, etc. Please clarify what is needed here. Related to point 1.

**Response 1.4**

We have corrected an error in the schematic related to this reviewer’s comment. Indeed, differential gene expression analysis follows the quantification of gene counts, and this has now been accurately represented in the updated diagram. Thank you for bringing this to our attention.

**Comment 1.5**

Please provide more information in the main text on how the bias was simulated.

**Response 1.5**

We thank the reviewer for bringing this to our attention. In addition to the detailed description of how we simulated annotational biases found in the Methods section, we have now included a brief overview of this procedure in the main text for better clarity and accessibility.

**Comment 1.6**

Please provide more information on where the ‘known and putative interactions between regulators and genes’ come from, and how this information is used in the tool.

**Response 1.6**

We thank the reviewer for this comment. In the manuscript we have delineated the distinction between known and putative interactions. We consider interactions to be ‘known’ if they are based on curated datasets and ‘putative’ if they are inferred from high-throughput assays.

**Comment 1.7**

I do not understand Figure 2E/B (the heatmap with green squares). Please provide more explanation of how it was built, what it means, and what we can conclude from it. Or perhaps just remove it. Similar for Figure 4B.

**Response 1.7**

We thank the reviewer for this comment.

The confusion about figure 3E most likely was caused by an insufficient captioning. We have edited the caption of this figure to make it more clear. We consider figure 3E important since it shows that the identified TFs control pathways related to neural and glial maintenance, as well as immune response. These observations support our claim that deregulation of these regulators is critical to AD. Figure 5B is also critical, since it illustrates the diverse mechanisms through which the identified RNA-binding proteins (RBPs) influence transcript fate.

**Comment 1.8**

Figure 5C: It is puzzling to me why the RBP regulon activity should be similar in healthy and AD shorter survival groups, but very different in AD longer survival. Can the authors provide some possible interpretation for this surprising result?

**Response 1.8**

Thank you for highlighting this point. Upon closer inspection, we indeed found it surprising that patients in the longer survival group exhibited increased activity of post-transcriptional regulons, while both donors from healthy and shorter survival groups had lower activity of these regulons. Our interpretation of these findings is that longer survival likely involves the activation of protective mechanisms that are not active under normal conditions. We have added this interpretation to the main text.

**Comment 1.9**

An extensive portion of the manuscript is spent describing how the pyPage tool is used to analyze Alzheimer’s disease datasets. It would be useful to include a comparison of results from traditional pathway analysis tools (e.g. GSEA, as described earlier in the text), to give the reader a sense of how different the results are using the new tool.

**Response 1.9**

We thank the reviewer for bringing this up. Following their suggestion, we compared regulons identified by pyPAGE, iPAGE, and Fgsea, focusing specifically on TF regulons due to the pronounced bias in their annotations noted in our previous analysis (S1A). Given the absence of a ground truth for this comparison, we concentrated on the robustness of the predictions. In this trial, pyPAGE performed favorably compared to iPAGE, identifying a robust set of transcriptional regulators. Similarly, Fgsea demonstrated high consistency across runs with ablated data. However, in contrast to pyPAGE, which identifies deregulation of only 3% of all TF regulons, Fgsea detects significant associations of over half (53%) of all TFs. Given recent studies (including ours), such massive deregulation of transcriptional programs is unlikely. Our previous studies of the pathological hallmarks of AD, specifically tau accumulation[^3^](https://www.zotero.org/google-docs/?CKi8fr) and microglia activation[^4^](https://www.zotero.org/google-docs/?pLuPYg), revealed that fewer than 10 out of the top 100 genes associated with these changes were transcription factors, underscoring that AD pathology is driven by the modulation of a limited number of key factors. This suggests that many of the Fgsea hits could be false positives, similarly to the results we have previously observed when benchmarking against synthetic data. Overall, the analysis highlights that pyPAGE is adept at identifying both robust and specific signals within the data.

**Minor:**

**Comment 1.10**

Define MSBB in introduction

**Response 1.10**

Thank you for this suggestion, we defined MSBB in the Introduction.

**Comment 1.11**

Define what you mean by biological regulon.

**Response 1.11**

Thank you for this suggestion. We defined the notion of a biological regulon and differentiated it from a biological pathway in the Introduction.

**Comment 1.12**

Figure 1E: Please clarify what input modulation means. Does 0.2 mean 20% of the full data? So does 1.0 mean full signal? If so, then why are the two curves different at 1? I would expect them to be the same with no subsampling or damping.

**Response 1.12**

Thank you for pointing it out. We have edited the caption of the figure 2C (previously 1E) explaining what input modulation is. The figure depicts the results of the analysis with two types of input modulation applied. In one case it is subsampling the number of genes in the input, in another it is reducing the deregulation signal. In the first cast would indeed mean that 0.2 is 20%. The curves have different values at 1.0 because they are run with different values of the parameter that controls the strength of the signal. In the first case it is set at 0.5, in the second it varies from 0.05 to 1.00.

**Comment 1.13**

Please add column labels to the heatmaps in Figures 2A, 3D, 4A,C

**Response 1.13**

We thank the reviewer for this comment. In these heatmaps, the columns correspond to equally populated gene bins sorted by their differential gene expression, making it unnecessary to label each column individually. Instead, to demonstrate that bins correspond to discretization of a continuous variable, we added arrows with captions indicating "Higher/lower expression/stability."

**Comment 1.14**

How were the cell types identified and annotated for the single cell analysis?

**Response 1.14**

We thank the reviewer for pointing out that this should be clarified. We have added to the Methods section that all of the datasets that we used were previously annotated by their authors.

**Comment 1.15**

Legend in Figure 2 is not consistent with panel labels. B is E, C is B, etc.

**Response 1.15**

We thank the reviewer for noticing this error. We have corrected this in the manuscript.

**Comment 1.16**

Figure 5B not referenced in text. P-value in 5B is different from what is reported in the table in 5D- should they be the same? If not, please clarify the difference.

**Response 1.16**

We thank the reviewer for pointing this out. Difference between the p-values is because in two cases we have used different models. In one case it was Kaplan–Meier (log rank) test and in the other Multivariate Cox model. We have clarified this in the paper.

**Comment 1.17**

Software comments:

Load_annotation appears to switch genes and pathways, using example data

I fixed it by setting ‘first_col_is_genes = True’, but should update her/his examples to be consistent with the data you provide

Similar comment for the gene conversion- did not work with arguments on the github

Please include list of dependencies/requirements with versions

**Response 1.17**

Thank you for highlighting these issues. Gene-set annotations typically follow this structure: Gene-set name, Gene1, Gene2, Gene3, etc. However, sometimes the gene-set name is listed first. In our example data, both types of annotations are present. We have now included a warning in the README to remind users to verify the structure of their annotation files and adjust the first_col_is_genes parameter accordingly.

Regarding the gene conversion issue you encountered, it seems to arise from using gene names in heatmaps, which is intended for situations where gene-sets are named after regulators, not pathway annotations as in their case. We've added a cautionary note about this in the documentation to prevent similar confusion. Furthermore, we have tested the example code in a new environment and confirmed it works correctly.

We have also included a file listing all dependencies and their versions to ensure compatibility and ease of setup.

**Reviewer #2**

**Comment 2.1**

Why to choose AD, especially considering that the iPAGE was developed by addressing cancers? Is the genetic risk landscape of AD that is different from cancers making an AD a unique challenge, more prune to biases? This shall be interesting to discuss, especially considering that most of the previous work were oriented to challenges posted by cancers, but what about AD, does it pose different challenges?

**Response 2.1**

We thank the reviewer for the comment. Our choice of Alzheimer's Disease (AD) as a case study primarily stems from our labs' focus on neuropathology (Kampmann + Goodarzi labs). In our view, AD is of interest as it presents unique challenges given that its progression involves the modulation of multiple gene programs in specific cell types. This complexity means that the deregulation signals presented by regulons in AD can be more subtle compared to those in monogenic diseases or even in some cancers, where a few key mutations can significantly drive the disease process. This nuance makes AD a particularly valuable and challenging case study for evaluating the effectiveness of our analytical methods in the context of multifactorial and multigenic disorders.

**Comment 2.2**

Along this line, How much customization need the pyPAGE be made to AD. Given any other diseases, is the method appliable and which hyperparameters need to be adjusted accordingly?

**Response 2.2**

We thank the reviewer for the comment. Despite Alzheimer’s disease being a condition with highly complex phenotype, our analysis didn’t require any customization of the program beyond changes that we introduced into the algorithm to mitigate annotational biases. Parameters that we used for running pyPAGE are listed in Table S4. One thing that we mention though is that because AD affects multiple brain regions, we aimed to include into our analysis multiple datasets in order to identify a robust set of regulatory signatures of the disease.

**Comment 2.3**

Pathway vs regulon: these two terms seem to be inter-changeably used but shall be clearly differentiated.

**Response 2.3**

We thank the reviewer for highlighting this important distinction, which was overlooked in our original draft. Generally, we define pathways as sets of functionally related genes, while regulons are groups of genes regulated by a common regulator. To clarify this, we have explicitly differentiated between these terms in the first paragraph of the Introduction and have carefully ensured their correct usage throughout the text. Though, in the benchmarking section these words can be used interchangeably (since in essence the method is agnostic to whether genes are functionally related or not), we stick to calling simulated gene-sets pathways.

**Comment 2.4**

In abstract, “unbiased way” is a really bold claim. Can authors really avoid all the biases.

**Response 2.4**

We thank the reviewer for bringing this up. Upon reflection, we agree that claiming pyPAGE provides completely unbiased results was overstated. The primary aim of pyPAGE is to mitigate the effects of annotational biases. And so any description of a method should be focusing only on this aspect. Consequently, we removed any references to pyPAGE being an “unbiased method.”

**Comment 2.5**

Along this line, authors claimed to be able to address the two biases, but it is not apparent to see how exactly this can be achieved.

First and foremost, I do not see why such what-authors-claimed-to-be “bias” is a bad thing, doesn’t this make more biological sense? Better annotated genes and abundant proteins are getting more weights. “differential expression of genes with specific functions provides stronger evidence for deregulation of its corresponding gene-sets than changes in expression of a more promiscuous gene.”

Second, if authors can convincingly address the above concerns, then we can talk about how exactly these two biases were tackled by proposed method. Using annotation bias as a covariate cannot completely address this type of bias and there is limitation. Besides, what about bias related to protein abundance difference? Authors shall really work out the details and explain to readers.

**Response 2.5**

We thank the reviewer for bringing this issue to our attention. Our work emphasizes that differential expression of genes with specific functions offers more robust evidence for the deregulation of corresponding gene-sets compared to changes in expression of genes that are involved in multiple pathways. And so, we posit that gene-set membership is an important covariate that should be taken into account when estimating gene-set enrichment. In other words, a gene that belongs to every gene-set will skew the underlying statistics if not accounted for. This is akin to cell-type identification in single cell data; genes that are universally and highly expressed across all cell types are not informative of cell type identity and are not included in cell type marker gene-sets; this is despite their high abundance in all cell types.

Furthermore, in the initial section of the Results, we demonstrate that gene-set annotations may exhibit inherent biases, as shown in Figure S1A. Specifically, these annotations deviate from the expected power-law distribution characteristic of scale-free networks. We attributed these biases to two factors: the protein's abundance and the extent of research attention the gene has received, as illustrated in Figure 1B. Although these factors are not biologically relevant, they can skew gene-set enrichment estimates if not properly accounted for. Other sources of bias may also exist beyond these.

To emphasize, in gene-set enrichment analysis, genes should not receive more weight as a function of their abundance or their centrality to human interest. Below is some added context as to how we view the source of these biases:

1. Protein abundance: mass-spectrometry forms the backbone of many protein-protein interaction maps that are used to define biological complexes. However, mass-spec has a limit of detection and more abundant and also “sticky” proteins tend to be picked up more commonly. This in turn results in gene-set annotations that are substantially more overlapping than it is biologically meaningful.
2. Technical artifacts: Similar to above, omics measurements have biases and artifacts as well that need to be accounted for but have not always been addressed. For example, the epigenome field has generated a “blacklist” of genomic regions where epigenomic tracks are not reliable. This is not the case for all approaches and many artifacts remain. For example, artifacts in CLIP assay for RNA-binding proteins can create overlapping regulons where the overlaps are not real and instead result from an underlying artifact of the assay.

**Comment 2.6**

In Fig 1B, authors shall report p-values along with spearman correlation coefficients, which suggest significance of such correlations. The same issue applies to Fig 2 C and D, Fig S6 as well as all other correlation results.

**Response 2.6**

We thank the reviewer for this comment. We have revised all figures that included correlation estimates and have now incorporated p-values into these figures (1B, 3C-D).

Similarly, we modified waterfall plots of correlations (3B and S6) and marked significant correlations with asterixis.

**Comment 2.7**

Any stats results that involve multiple times of tests must be corrected for multiple-testings, due to the fact that they are prune to false positives. P<0.05 is not enough, and this issue applies to, but not limited to, Fig 2E, Fig 3CD.

**Response 2.7**

We thank the reviewer for this comment. For figures 3E, 4C, 5D S4, S8, 4E we have used adjusted p-values.

**Comment 2.8**

“physical interactions for a given protein correlates with this protein abundance” and authors believed that this indicates structural biases. why this is the case? For sure, you can raise a few exceptions, but how can you conclude for sure that the majority shall not correlate?

**Response 2.8**

We thank the reviewer for this comment. In the original draft, we demonstrated that protein abundance correlates with the number of recorded interactions for a given protein (Figure 1B). There isn’t an existing paradigm that supports that more abundant proteins should interact with more partners. Instead, this correlation likely arises due to the fact that more abundant proteins are more likely to be detected in interaction assays, due to the detriment of their less abundant proteins. This does not negate the biological relevance of these interactions, but it does indicate a bias that may affect the analysis. To emphasize, this is a demonstration how biases may arise in annotations, but not the only way.

**Comment 2.9**

What do you mean by multiple data modalities? Regardless of TF, RBP, and miRNA, aren’t they all table formats showing abundance information?

**Response 2.9**

We thank the reviewer for the comment. To clarify, the term "multiple data modalities" refers to our model's capability to process different types of input data, ranging from dense bulk data to sparse single-cell sequencing data. We have made this clearer in the revised text.

**Comment 2.10**

In Fig 5D, it is commendable to see the hint that stratification by regulon works, but the fact that cluster B seems to have greater variance may indicate that there is still significant heterogeneity leftover, therefore authors may try to break them into finer subgroups. Could it produce even better stratification?

**Response 2.10**

We thank the reviewer for their suggestion. We have performed further subclustering of samples in Group B (with lower activity), dividing them into two subgroups with low and moderate activity. We then applied the Kaplan-Meier model to these subgroups and observed that samples with moderate activity exhibit significantly higher survival rates (Figure S9D). We have avoided subclustering of the group A, because then there would be too few samples in the subclusters, which results in loss of statistical power.

**Comment 2.11**

The author compared the performance of pyPAGE, iPAGE, and fgsea on each simulated dataset. However, their performance on real-world data is still unclear and shall be further evaluated, and importantly against other popular enabling tools of the same kind.

**Response 2.11**

We thank the reviewer for bringing this up. Following their suggestion, we compared regulons identified by pyPAGE, iPAGE, and Fgsea, focusing specifically on TF regulons due to the pronounced bias in their annotations noted in our previous analysis (S1A). Given the absence of a ground truth for this comparison, we concentrated on the robustness of the predictions. In this trial, pyPAGE performed favorably compared to iPAGE, identifying a robust set of transcriptional regulators. Similarly, Fgsea demonstrated high consistency across runs with ablated data. However, in contrast to pyPAGE, which identifies deregulation of only 3% of all TF regulons, Fgsea detects significant associations of over half (53%) of all TFs. Given recent studies (including ours), such massive deregulation of transcriptional programs is unlikely. Our previous studies of the pathological hallmarks of AD, specifically tau accumulation[^3^](https://www.zotero.org/google-docs/?1UqiYi) and microglia activation[^4^](https://www.zotero.org/google-docs/?Eq7137), revealed that fewer than 10 out of the top 100 genes associated with these changes were transcription factors, underscoring that AD pathology is driven by the modulation of a limited number of key factors. This suggests that many of the Fgsea hits could be false positives, similarly to the results we have previously observed when benchmarking against synthetic data. Overall, the analysis highlights that pyPAGE is adept at identifying both robust and specific signals within the data.

**Comment 2.12**

Also, when comparing against other methods, data shall be split into training set and testing set, and preferably bootstrap this split process for a large number of times and only report performance indices (with confident intervals) based on testing dataset.

**Response 2.12**

We thank the reviewer for this comment. In response to this issue, we want to point out that splitting the data into training and testing sets is relevant to test performance of the models with learnable parameters. However, since pyPAGE operates differently by first deterministically computing conditional mutual information and then performing statistical estimation of its significance, training and testing data on different data splits does not apply to it.

**Comment 2.13**

In the analysis of single-cell data, for the differential expression of each cell type-specific gene, the author used estimated differential gene expression between two groups of samples. Would using marker genes specific to each cell type be more appropriate?

**Response 2.13**

We thank the reviewer for this question. As demonstrated by the robustness test (Figure 2C), pyPAGE performs optimally when provided with a larger amount of genes in the input. Thus limiting the analysis to only a few marker genes would compromise its ability to accurately estimate conditional mutual information and assess its significance.

**Comment 2.14**

The author explored patterns in the single-cell data that had remained hidden in their analysis of bulk RNA-seq. Currently, spatial transcriptomics data related to Alzheimer’s disease have also been released, and I suggest considering adding an analysis or discussing relevant matters that specifically focused on spatial transcriptomics data. This way, it would encompass the mainstream transcriptional data involving gene-set enrichment analysis.

**Response 2.14**

We thank the reviewer for suggesting exploring spatial datasets. Following it, we have incorporated into our manuscript an analysis of middle temporal gyrus spatial sequencing data from the Visium platform. For this data, we estimated differential TF activity in various cortical layers (Figure 4E).This analysis showed that most of the TF regulons exhibit similar regulation patterns across cortical layers. We have not been able to perform the analysis of RBP or microRNA regulons, since there are no available tools for the estimation of transcript stability in spatial data.

**Comment 2.15**

Some sentences need to be rephased, because it is hard to follow:

For example, the observation that a protein that is only annotated as part of a few gene sets is differentially expressed narrows down the selection of potentially relevant pathways much more than a similar observation about a protein that is present in many gene sets.

the number of gene sets a gene is assigned to varies significantly among all genes.

which are by and large ignored

**Response 2.15**

We are thankful to the reviewer for pointing out that some of the sentences sound ambiguous. In response to the reviewer's feedback, we have revised sentences that were previously difficult to understand. In addition the text was reviewed by our editor.

**Reviewer #3**

**Comment 3.1**

At its core, pyPAGE is based on conditional mutual information (CMI). I would suggest adding the definition/formula of CMI in the methods section. (As a minor issue, CMI is used in the text before defining it). Is any smoothing applied while estimating the probabilities and conditional probabilities used to compute CMI?

**Response 3.1**

We thank the reviewer for this question. Following the reviewer’s suggestion we added a formula for conditional mutual information into the main text. We also specify in the text that no smoothing of probabilities is performed for computing conditional mutual information.

**Comment 3.2**

The filtering procedure addresses the issue of only considering regulators whose misregulation cannot be explained by previously accepted regulators. These thoughts bring me to the following list of suggestions:

• Expand the description of the filtering procedure by adding motivation.

• Is the filtering procedure essential for the good performance of pyPAGE.

**Response 3.2**

We thank the reviewer for this comment. In the manuscript we expand the description of how filtering works. For each gene-set in the list of most informative gene-sets, we compute the following ratio:

$\frac{I(candidate gene-set; expression| accepted gene-set)}{I(candidate gene-set; accepted gene-set)}$

which measures how much information each gene-set adds. We require this ratio to be greater than some fixed threshold which is a hyperparameter of the model (the values that we used in the experiment are provided in Table S4). Motivation behind this is that in the final output should be a representative set of gene programs (regulons), where each new entry adds a significant amount of information to the information that is already present in the selected ones.

The utility of this filtering is not solely in distinguishing overlapping regulons; in case of hierarchical annotations, like GO terms, where gene-sets are highly redundant and overlapping, this approach substantially reduces clutter and reveals the major representative gene-sets to focus on.

Though filtering gene-sets in our output is an important aspect of our pipeline, it is not a standalone solution for addressing annotation biases. In order to effectively employ this procedure, the selection of gene-sets to which filtering is applied should be formed in an unbiased manner. Additionally, we should point out that this filtering had already been introduced in the original version of iPAGE[^1^](https://www.zotero.org/google-docs/?SuD6Y6) and still pyPAGE shows improvement over it both with simulated and real data.

**Comment 3.3**

Clearly state if and where (evaluation, AD applications, etc.) it is used and with what r.

**Response 3.3**

We thank the reviewer for the question. In some cases a user might want to consider the whole space of significant hypotheses and not only the significant ones. In the text we added a description in which cases filtering was used (Table S4).

**Comment 3.4**

The generation of synthetic data that mimics the hypothesized effects of biases in the annotation database seems convoluted and arbitrary. But after spending a few days trying to suggest something better, I gave up. The described procedure does generate datasets that make the performance of the pyPAGE better than that of iPAGE. This is not surprising, as pyPAGE directly accounts for the simulated biases, but tt the very least, that shows that pyPAGE’s implementation is likely correct. I think the paper will have a much stronger argument if the authors also compare pyPAGE’s and iPAGE’s results on a real dataset. The expectation is that iPAGE will generate more significant regulators. It would be reassuring if the authors could show that the extra ones are artifacts caused by the annotation biases.

**Response 3.4**

We thank the reviewer for bringing this up. Following their suggestion, we compared regulons identified by pyPAGE, iPAGE, and Fgsea, focusing specifically on TF regulons due to the pronounced bias in their annotations noted in our previous analysis (S1A). Given the absence of a ground truth for this comparison, we concentrated on the robustness of the predictions. In this trial, pyPAGE performed favorably compared to iPAGE, identifying a robust set of transcriptional regulators. Similarly, Fgsea demonstrated high consistency across runs with ablated data. However, in contrast to pyPAGE, which identifies deregulation of only 3% of all TF regulons, Fgsea detects significant associations of over half (53%) of all TFs. Given recent studies (including ours), such massive deregulation of transcriptional programs is unlikely. Our previous studies of the pathological hallmarks of AD, specifically tau accumulation[^3^](https://www.zotero.org/google-docs/?CY69Bl) and microglia activation[^4^](https://www.zotero.org/google-docs/?OTs8Em), revealed that fewer than 10 out of the top 100 genes associated with these changes were transcription factors, underscoring that AD pathology is driven by the modulation of a limited number of key factors. This suggests that many of the Fgsea hits could be false positives, similarly to the results we have previously observed when benchmarking against synthetic data. Overall, the analysis highlights that pyPAGE is adept at identifying both robust and specific signals within the data.

**Comment 3.5**

Many annotation datasets of regulators and their targets and bulk and single-cell RNASeq datasets are used. Basic information about all these datasets is missing. For all annotation databases, it is essential to present the number of regulators, the number of targets, and the distributions of the number of regulators per gene. For the RNASeq datasets, the authors should include the number of samples, control, and cases in the original dataset, the one used for the analysis after filtering, and a brief explanation about why the dataset is used.

**Response 3.5**

We thank the reviewer for this comment. We have updated the Methods section to include detailed information about each dataset utilized in our study. As requested we now provide the number of samples, including control and case numbers, both in the original and the filtered datasets used for analysis. Additionally, we have summarized the key information regarding the annotation databases, including the number of regulators, the number of targets, and the distribution of regulators per gene, in Table S3.

**Comment 3.6**

I find the pipeline shown in Figure 1 C misleading: (1) It does not reflect the presented experiments. (2) What is gene count? (3) It seems the pyPAGE jointly analyzes DE and differential transcript stability while these are analyzed independently. I’d suggest showing a diagram or a table listing all experiments discussed in the results. The table should list the annotation database, RNASeq data, and the measure used for each experiment…

It is also important to be specific about the parameters used for the analyses. How many bins are used? What is the r for the additional filtering? What are the detailed versions and parameters of the tools used to compute the differential measures from the RNASeq datasets?

**Response 3.6**

We thank the reviewer for the feedback. We have revised the pipeline schematic in Figure 2A to more accurately reflect the experiments presented, separating it into two branches: transcriptional regulation and post-transcriptional regulation. Additionally, we have included Table S4, which summarizes all the experiments, detailing the annotation databases, RNASeq data, and the measures used for each experiment. This update should clarify the methodologies and enhance the transparency of our analyses.

**Comment 3.7**

It is unclear why the BM36 region is used for the bulk RNASeq analysis.

**Response 3.7**

We thank the reviewer for the feedback. We selected the BM36 region for our initial bulk RNASeq analysis because it's notably vulnerable in Alzheimer's disease, often showing early neurodegenerative changes[^5^](https://www.zotero.org/google-docs/?yojzle). To validate and expand our findings, we also analyzed single-cell RNA sequencing data from the human prefrontal cortex and spatial transcriptomics data from the middle temporal gyrus. This multi-regional approach ensures the robustness of inferred regulatory signatures, highlighting their relevance to Alzheimer's pathology. Following this reviewer’s comment, we have added in text motivation for using each dataset.

**Comment 3.8**

Can the DE be used with the RBPs and microRNAs instead of the differential RNA stability?

**Response 3.8**

We thank the reviewer for the comment. In principle, DE can be used for analysis of RBP and miRNA regulons; however, in practice, DE is much more heavily influenced by transcriptional regulatory programs rather than post-transcriptional regulons. Focusing the analysis on differential RNA stability, allows us to sharpen the contribution of these regulons and increases the sensitivity of the approach to pick up these regulatory programs. We have discussed this in the original REMBRANDTS paper as well[^2^](https://www.zotero.org/google-docs/?1HYdks). In other words, since these regulators primarily influence RNA stability, differential RNA stability is a more relevant measure for this context.

**Comment 3.9**

Why does the single-cell RNA analysis of the RPBs and microRNAs use differential splicing and deregulation measures instead of the differential RNA stability as done in the bulk RNASeq analysis?

**Response 3.9**

We thank the reviewer for the question. We used differential splicing and deregulation measures in the single-cell RNA analysis of RBPs and microRNAs due to the absence of tools suitable for estimating RNA stability in single-cell data. Instead, we focused on other aspects of RNA metabolism that could be analyzed with available methods. The motivation is stated in the text. To reiterate, REMBRANDTS requires high confidence estimation of pre-mRNA and mRNA levels, which rely on intronic counts. Single cell RNA sequencing is fundamentally a 3’-end sequencing approach, and while some intronic regions are picked up due to the presence of endogenous poly A tracts, and even used in tools like RNA velocity, they do not provide sufficient coverage for REMBRANDTS analysis. Therefore, with current technologies, single cell RNA sequencing is not sufficient to deconvolve transcriptional and post-transcriptional changes in gene expression.

**Comment 3.10**

Why is a different dataset used to demonstrate the heterogeneity of AD patients?

**Response 3.10**

We thank the reviewer for the comment. We have used different datasets to analyze the heterogeneity of AD to ensure the robustness and generalizability of our findings. Relying on a single RNA-seq dataset for both gene-set enrichment and survival analyses could introduce biases linked specifically to that dataset, potentially skewing the results and limiting their applicability to other populations or brain regions.

**Comment 3.11**

I don’t quite understand how the pyPAGE’s CMI output is used to measure the activity of a regulon in an individual. As the split of the AD patients into two groups is a major result, the procedure should be made explicit in the methods.

**Response 3.11**

We thank the reviewer for the feedback. Following their request, we have detailed this process in the text. Briefly, we normalized gene expression across samples and used these values along with pyPAGE's estimates of conditional mutual information to quantify the activity of TF regulons in each individual. For RNA-binding proteins (RBPs) and microRNAs, we utilized tools like Salmon and REMBRANDTS to estimate transcript stability in each sample. We then applied normalized stability values to infer the activity of RBP and miRNA regulons. This approach allows us to assess regulon activity on a per-sample basis using normalized expression and stability values instead of differential metrics.

**Minor**

**Comment 3.12**

“… genes themselves, that underlies cellular pathologies” -> “… genes themselves, that underly cellular pathologies.”

**Response 3.12**

We thank the reviewer for pointing out this error. It has been corrected.

**Comment 3.13**

What does “and uses an underlying deterministic statistic rather than permutation-based approaches” mean? Aren’t the p-values in pyPAGE based on non-paramemtric permutation?

**Response 3.13**

We thank reviewer for bringing this to our attention. You are right; our previous description may have caused some confusion. While pyPAGE calculates conditional mutual information (CMI) deterministically to assess relationships, it also incorporates a non-parametric permutation approach for determining the statistical significance of these CMI values. This method involves randomly permuting data vectors and calculating p-values to evaluate the likelihood of observing such CMI measurements by chance, thereby combining both deterministic and permutation-based methodologies. We have updated the text, removing any reference to pyPAGE as a deterministic method.

**Comment 3.14**

What are the typical bin numbers used for differential expression and the gene set membership profiles?

**Response 3.14**

We thank the reviewer for this question. Differential expression is typically quantized into 10 bins, and gene-set membership profiles are divided into 3 bins. These default values were used for our benchmarking and analysis of Alzheimer's disease, as detailed in newly added Table S6.

**Comment 3.15**

In the left column of Figure 2A, a direction should be indicated. I.e., yellow means the TF has an increased expression in AD. How are the enrichment scores for the bins computed?

**Response 3.15**

We thank the reviewer for this question. We have added a detailed explanation to the Methods section. To quantify the level of over- and under-representation in each bin, we employ the hypergeometric test to calculate two distinct p-values: one for the likelihood of observing fewer genes from the same gene-set in a given bin (under-representation), and another for the probability of observing more genes (over-representation). If the p-value for over-representation is lower than that for under-representation, we consider the gene-set to be over-represented in that bin; otherwise, it is considered under-represented. The minimal p-value is then log-transformed, and multiplied by -1 if the gene-set is over-represented.

**Comment 3.16**

Figure 2E. Are the TF functions identified using only the subset of the regulon targets exhibiting DE or all targets?

**Response 3.16**

We thank the reviewer for this question. We have clarified this in the text. We analyze all targets of the regulons to identify the functions of the transcription factors, not just the subset exhibiting differential expression. This comprehensive approach allows for a more complete understanding of the transcription factors' roles and interactions.

**Comment 3.17**

The legend of Figure 2 is not in sync with the figure. The panel labels are permuted.

**Response 3.17**

We thank the reviewer for pointing this out. We have corrected the panel labels in the legend of Figure 3 to ensure they match the figure accurately.

**Comment 3.18**

I would suggest splitting Figure 1: panels A and B, focusing on the properties of gene annotations and the rest, focusing on the pyPAGE and its evaluation.

**Response 3.18**

We thank the reviewer for this comment. As you suggested, we have split Figure 1 into two separate figures.

**Comment 3.19**

Tables S1 and S2 that summarize the main results of the AD analysis seem very useful. Can you promote it to the main text?

**Response 3.19**

We thank the reviewer for the feedback. As you suggested, we have moved Tables 1 and 2, which summarize the main results of the Alzheimer's disease analysis, into the main text for better accessibility and visibility.

**References**

[(1)	Goodarzi, H.; Elemento, O.; Tavazoie, S. Revealing Global Regulatory Perturbations across Human Cancers. *Mol. Cell* **2009**, *36* (5), 900–911. https://doi.org/10.1016/j.molcel.2009.11.016.](https://www.zotero.org/google-docs/?MZL9fS)

[(2)	Alkallas, R.; Fish, L.; Goodarzi, H.; Najafabadi, H. S. Inference of RNA Decay Rate from Transcriptional Profiling Highlights the Regulatory Programs of Alzheimer’s Disease. *Nat. Commun.* **2017**, *8* (1), 909. https://doi.org/10.1038/s41467-017-00867-z.](https://www.zotero.org/google-docs/?MZL9fS)

[(3)	Samelson, A. J.; Ariqat, N.; McKetney, J.; Rohanitazangi, G.; Bravo, C. P.; Goodness, D.; Tian, R.; Grosjean, P.; Abskharon, R.; Eisenberg, D.; Kanaan, N. M.; Gan, L.; Condello, C.; Swaney, D. L.; Kampmann, M. CRISPR Screens in iPSC-Derived Neurons Reveal Principles of Tau Proteostasis. *bioRxiv* **2023**, 2023.06.16.545386. https://doi.org/10.1101/2023.06.16.545386.](https://www.zotero.org/google-docs/?MZL9fS)

[(4)	Dräger, N. M.; Sattler, S. M.; Huang, C. T.-L.; Teter, O. M.; Leng, K.; Hashemi, S. H.; Hong, J.; Aviles, G.; Clelland, C. D.; Zhan, L.; Udeochu, J. C.; Kodama, L.; Singleton, A. B.; Nalls, M. A.; Ichida, J.; Ward, M. E.; Faghri, F.; Gan, L.; Kampmann, M. A CRISPRi/a Platform in Human iPSC-Derived Microglia Uncovers Regulators of Disease States. *Nat. Neurosci.* **2022**, *25* (9), 1149–1162. https://doi.org/10.1038/s41593-022-01131-4.](https://www.zotero.org/google-docs/?MZL9fS)

[(5)	Wang, M.; Roussos, P.; McKenzie, A.; Zhou, X.; Kajiwara, Y.; Brennand, K. J.; De Luca, G. C.; Crary, J. F.; Casaccia, P.; Buxbaum, J. D.; Ehrlich, M.; Gandy, S.; Goate, A.; Katsel, P.; Schadt, E.; Haroutunian, V.; Zhang, B. Integrative Network Analysis of Nineteen Brain Regions Identifies Molecular Signatures and Networks Underlying Selective Regional Vulnerability to Alzheimer’s Disease. *Genome Med.* **2016**, *8* (1), 104. https://doi.org/10.1186/s13073-016-0355-3.](https://www.zotero.org/google-docs/?MZL9fS)
